# Characterization of Urinary Pesticide Metabolite Concentrations of Pregnant Women in Suriname

**DOI:** 10.3390/toxics10110679

**Published:** 2022-11-10

**Authors:** Cecilia S. Alcala, Maureen Y. Lichtveld, Jeffrey K. Wickliffe, Wilco Zijlmans, Arti Shankar, Ellen Rokicki, Hannah Covert, Firoz Z. Abdoel Wahid, Ashna D. Hindori-Mohangoo, Alies van Sauers-Muller, Carmen van Dijk, Jimmy Roosblad, John Codrington, Mark J. Wilson

**Affiliations:** 1 Department of Environmental Medicine and Public Health, Icahn School of Medicine at Mount Sinai, New York, NY 10029, USA; 2 Environmental and Occupational Health, University of Pittsburgh School of Public Health, Pittsburgh, PA 15261, USA; 3 Environmental Health Sciences, University of Alabama at Birmingham, Birmingham, AL 35233, USA; 4 Faculty of Medical Sciences, Anton de Kom University of Suriname, Paramaribo, Suriname; 5 Department of Biostatistics and Data Science, Tulane University School of Public Health and Tropical Medicine, New Orleans, LA 70112, USA; 6 Department of Environmental Health Sciences, Tulane University School of Public Health and Tropical Medicine, New Orleans, LA 70112, USA; 7 Scientific Research Center Suriname, Academic Hospital Paramaribo, Paramaribo, Suriname; 8 Foundation for Perinatal Interventions and Research in Suriname (Perisur), Paramaribo, Suriname; 9 Pesticide Division, Ministry of Agriculture, Animal Husbandry, and Fisheries of Suriname, Paramaribo, Suriname; 10 Clinical Chemical Laboratory, Academic Hospital Paramaribo, Paramaribo, Suriname

**Keywords:** pesticides, agriculture, prenatal exposure, Suriname, biomonitoring, metabolites

## Abstract

Prenatal exposure to pesticides and the association with adverse health outcomes have been examined in several studies. However, the characterization of pesticide exposure among Surinamese women during pregnancy has not been assessed. As part of the Caribbean Consortium of Research in Environmental and Occupational Health research program, 214 urine samples were collected from pregnant women living in three regions in Suriname with different agricultural practices: capital Paramaribo, the rice producing district Nickerie, and the tropical rainforest, the Interior. We used isotope dilution tandem mass spectrometry to quantify urinary concentrations of biomarkers of three pesticide classes, including phenoxy acid herbicides and organophosphate and pyrethroid insecticides, all of which are commonly used in agricultural and residential settings in Suriname. We observed that participants residing in Nickerie had the highest urinary metabolite concentrations of 2,4-dichlorophenoxyacetic acid and pyrethroids compared to those from Paramaribo or the Interior. Paramaribo had the highest concentrations of organophosphate metabolites, specifically dialkyl phosphate metabolites. Para-nitrophenol was detected in samples from Paramaribo and the Interior. Samples from Nickerie had higher median urinary pesticide concentrations of 2,4-dichlorophenoxyacetic acid (1.06 μg/L), and the following metabolites, 3,5,6-trichloro-2-pyridinol (1.26 μg/L), 2-isopropyl-4-methyl-6-hydroxypyrimidine (0.60 μg/L), and 3-phenoxybenzoic acid (1.34 μg/L), possibly due to residential use and heavy rice production.

## 1. Introduction

Agriculture is a growing sector in Suriname, as it accounts for 5% of the country’s foreign exchange earnings and for employing 17% of the population [1,2]. Nickerie is the main agricultural district located in the northwest region of Suriname. This district is well known for its large rice and banana production and for export to the Caribbean region and countries in the European Union [3,4]. The most predominantly used pesticides in Nickerie are the following: insecticides-: malathion, diazinon, cypermethrin, chlorpyrifos; fungicides: mancozeb; herbicides: 2,4-D dimethyl amine salt, paraquat; and molluscides: fentin acetate [5]. In addition to using pesticides for agricultural purposes, they are also used for residential purposes and to control for vector borne diseases across the country [6]. In Paramaribo, the capital city of Suriname, and the Interior regions, the herbicide, paraquat and the insecticide, malathion are frequently used for banana and agricultural production [5]. According to the Ministry of Agriculture, Animal Husbandry, and Fisheries, the import of pesticides into Suriname has increased over the past 26 years [5]. Pregnant women and children can be exposed to pesticides through several environmental media (air, food, surfaces, and water), and through multiple routes of exposure (dermal, ingestion, breastmilk, and inhalation) [7,8,9,10]. Furniture, carpets, and items at home can also act as repositories for parent compounds and their metabolites [11].

Pesticide products comprise both active and inert ingredients. Active ingredients in pesticides are important in reducing or eliminating a broad range of animal, plant, and fungal pests [12]. Inert ingredients are essential for product performance and usability [13]. The active ingredients of pesticides are described and categorized by the types of pests they control or how they work. Pesticides are grouped into specific groups depending on the types of pests they kill. The most common groups of pesticides include: insecticides: kills insects and other arthropods; herbicides: kills weeds and other unwanted plants; rodenticides: controls for mice and other rodents; fungicides: kills fungi; repellents: repels pests, which also includes insects and birds; nematicides: kills nematodes; molluscicides: kills snails and slugs; miticides: kill mites that feed on plants and animals; and algicides: kills algae in lakes, canals, and multiple other sites [12].

Phenoxy acid herbicides, organophosphate insecticides, and pyrethroid insecticides are commonly used throughout Suriname and exposure could result in adverse health outcomes, specifically in vulnerable populations including pregnant women and children. There are numerous factors that determine potential health outcomes, including the type(s) of pesticide(s), the duration, frequency, and the route of exposure, and the health status of the individual [14]. Pesticides can bioaccumulate in humans (i.e., stored in body fat) and can be metabolized and excreted [15,16,17]. The mode of action varies based on the chemical group. Organophosphate and carbamate insecticides inhibit the acetylcholinesterase (AChE) enzyme in the nervous system [18,19]. Pyrethroids delay sodium channels from opening and closing [19,20,21]. This results in uncontrolled nerve firing which causes convulsing tremors and shaking [19,22]. For the phenoxy acid herbicides, the mode of action is through uncontrolled auxin growth activation [23]. These non-persistent chemicals have short half-lives. The pyrethroid insecticide, 3-phenoxy benzoic acid, persists in fatty tissues and has a half-life of 4–5 days [18]. Organophosphate insecticides are metabolized quickly, and some have half-lives up to 3 days in humans [18]. The half-life of phenoxy acid herbicides, like 2,4-dichlorophenoxyacetic acid, is 10–20 h [18,24].

There has been extensive research on pesticide exposures and their associations with acute and chronic adverse health outcomes. Exposure to pesticides may result in detrimental health effects, including in pregnant women and children [25]. Pregnant women’s exposure to pesticides is a public health concern due to the rapid development of infant organ systems during the prenatal period [26]. Exposure to pesticides during pregnancy can lead to adverse birth outcomes, including low birth weight, preterm birth, perinatal asphyxia, congenital anomalies, and developmental deficits [27,28,29,30]. Some studies have found an association between prenatal exposure to phenoxy acid herbicides and adverse health outcomes in infants and children, specifically of 2,4-dichlorophenoxyacetic acid (2,4-D) [31,32,33]. These impacts include reduced auditory processing [32], shorter anogenital distance in 3 month old boys [31], and hay fever allergy development [33]. Organophosphate insecticides (OPs) are potent toxicants that target the nervous systems of insects and other pests in addition to vertebrates including humans [34,35]. Pregnant women’s exposure to organophosphate insecticides is associated with adverse child development outcomes, including impaired cognition [36,37,38], attention deficit hyperactivity disorder (ADHD) [35,39], impaired concentration, slowed information processing and motor functioning, anxiety, confusion, tremors, seizure, and autism spectrum disorders [35,40,41]. Pyrethroids are metabolized quickly in the body and most degrade rapidly in sunlight [40]. Multiple studies have assessed the association between prenatal exposures to pyrethroids and adverse health outcomes, however, the results are inconsistent [31,41,42,43,44,45].

In Suriname, pesticide residues of insecticides, including two banned organochlorines (endosulfan and lindane), were present in produce in excess of the maximum residue levels permissible in the European Union [46]. However, the characterization of pesticide exposure among pregnant women in Suriname still remains a research gap, as is biomonitoring of pesticides. As an important step towards filling this research gap, we examined urinary pesticide metabolite concentrations in pregnant women residing in the following regions in Suriname: Paramaribo, Nickerie, and the Interior. Study participants were enrolled in the Caribbean Consortium for Research in Environmental and Occupational Health (CCREOH), an environmental epidemiology cohort study ongoing in the country since 2015 [47]. We hypothesized that exposure to pesticides used in commercial agriculture will be higher in women residing in Nickerie, and pesticides used in residential settings will be higher in women residing in Paramaribo.

## 2. Materials and Methods

### 2.1. Cohort Characteristics and Inclusion Criteria

CCREOH was established to examine high priority environmental and occupational health risks likely linked to poor perinatal and neonatal health outcomes. The environmental epidemiology cohort study recruited 1200 Surinamese mother/child dyads from December 2016 to July 2019.

Participants of this study (*n* = 214) were enrolled in the cohort from three regions: (1) Paramaribo, the urban capital city with a dense population; (2) Nickerie, a semi-rural area known for its rice and banana production; and (3) the Amazonian Interior, a largely undeveloped rural area with the lowest population density. The inclusion criteria consisted of pregnant women recruited during their first or second trimester from the following facilities for prenatal services in Suriname: the Academic Hospital Paramaribo, Diakonessenhuis Hospital, ‘s-Lands Hospital, St. Vincentius Hospital, Mungra Medical Centre Nickerie, the Regional Health Department Clinics, and the Medical Mission Primary Health Care Suriname clinics in the Interior [47]. The women were 16 years or older, had a single gestation, planned delivery at one of the study hospitals, prenatal clinics or midwife facilities associated with those hospitals or clinics, and provided informed consent/assent. For the purposes of this study, we included all participants with completed urine samples during the first or second trimester and that had complete demographic data (*n* = 214).

Pregnant women who met the inclusion criteria were identified by their treating physician, mid-wife, or health assistant during their regular prenatal visits. Once identified, they were asked to participate in the study by a trained study assistant or the physician. All participants provided informed written consent (aged 18+) or assent and parental permission (aged 16–17). After consent was obtained, urine samples were collected from the participants. Participants were given $20.00 to compensate for their time. Further information of the study design and inclusion and exclusion criteria of the CCREOH cohort can be found in Zijlmans et al. [47].

### 2.2. Urine Sample Collection

Each participant was assigned a sterile urine collection cup with a tamper sealed lid, polypropylene vials, and barcoded labels. Participants were instructed to wash their hands with only water and air dry, not to remove the cap from the cup until ready to void, collect at least 60 mL urine in the cup, not to touch the inside of the cup at any time, replace the screw cap and tighten to avoid leakage, and place the label with participant ID on the side of the cup. Individual zip bags were provided for sample storage. Collected samples were refrigerated immediately. Then, within 4 h of collection, the urine was aliquoted into 10 mL polypropylene cryovials, labeled, frozen at −20 °C, and shipped overnight on dry ice to the U.S. Centers for Disease Control and Prevention’s (CDC) Environmental Health Laboratory.

### 2.3. Laboratory Analysis

Urine samples were analyzed by the CDC Environmental Health Laboratory for three pesticide classes including a phenoxy acid herbicide: 2,4-dichlorophenoxyacetic acid; ten organophosphate insecticide metabolites: malathion dicarboxylic acid, 3,5,6-trichloro-2-pyridinol, 2-isopropyl-4-methyl-6-hydroxypyrimidine, para-nitrophenol, diethyldithiophosphate, diethylphosphate, dimethyldithiophosphate, dimethylphosphate, dimethylthiophosphate, and diethylthiophosphate, and three pyrethroid insecticide metabolites: 4-fluoro-3-phenoxybenzoic acid, 3-phenoxybenzoic acid, and trans-3-(2,2-Dichlorovinyl)-2,2-dimethylcyclopropane carboxylic acid, shown in Table 1.

A modification of a solid phase extraction high performance liquid chromatography- isotope dilution tandem mass spectrometry (SPE-HPLC-MS/MS) method was performed on an Agilent 1100 (Agilent Technologies) and was used to analyze urine samples for six dialkyl phosphate metabolites: dimethylphosphate, diethylphosphate, dimethylthiophosphate, dimethyldithiophosphate, diethylthiophosphate, and diethyldithiphosphate [48,49]. A semi-automated SPE-HPLC-MS/MS method was also used to analyze samples for four specific metabolites of organophosphate insecticides: 3,5,6-trichloro-2-pyridinol, 2-isopropyl-4-methyl-6-hydroxypyrimidine, para-nitrophenol, and malathion dicarboxylic acid; three metabolites of pyrethroids: 4-fluoro-3-phenoxybenzoic acid, 3-phenoxybenzoic acid, and trans-3-(2,2-dichlorovinyl)-2,2-dimethylcyclopropane carboxylic acid; and the herbicide 2,4-dichlorophenoxyacetic acid [50].

To collect the specific gravity measurements for each urine sample, 10 mL of urine specimens were mixed and transferred to disposable test tubes at the Clinical Chemistry Laboratory at the Academic Hospital Paramaribo. Each test tube was loaded onto a UriScan Super from YD Diagnostics. The sample codes were manually entered into the machine. Specific gravity was measured by a built-in refractometer. The results were uploaded to the laboratory information system and then copied to an Excel file. We collected both fresh and frozen specific gravity measurements. Fresh specific gravity measurements on 45% of the participants. As a result, frozen specific gravity measurements were collected for all 214 participants. We compared fresh vs. frozen specific gravity measurements.

### 2.4. Statistical Analysis

All statistical analyses were conducted using IBM SPSS Statistics Version 26 and RStudio (Armonk, NY, USA) (IBM, 2019). We imputed pesticide metabolite concentrations below the limit of detection for the statistical analysis. The log-ratio expectation-maximization (EM) algorithm function based on compositional data was used to impute non-detectable concentrations under a Bayesian paradigm. This function uses model-based ordinary and robust EM algorithms to impute left-censored values using coordinates representative of compositional data sets. The imputations were conducted using the zCompositions package in R [51]. Specific gravity (*SG*) adjustments were made on the pesticide metabolite concentrations to account for fluctuations in urine dilution using a reference specific gravity measurement of 1.02 ug/L. The following equation was used, based on the *SG* reference (*SG_ref_*) value:

(1) $$C_{corr}=\frac{C_{i}\left(SG_{ref}-1\right)}{\left(SG-1\right)}$$

*C_corr_* = Corrected concentration;

*C_i_* = Measured concentration of the biological indicator;

*SG* = Participant-specific specific gravity measurements; and

*SG_ref_* = Reference specific gravity measurement [51].

To describe the study cohort, we presented descriptive characteristics of the population at enrollment, which included age, education level, ethnicity, marital status, and household income. Descriptive statistics of the urinary pesticide metabolite concentrations after imputations, included the median, standard deviation, and percentiles were used for pesticides with a large percentage of non-detects.

The Shapiro–Wilk test was conducted to assess the normality of all pesticide metabolites with successful imputations. The assumptions for normality were not met, *p* < 0.05. We used non-parametric tests for further testing of specific-gravity adjusted concentrations, specifically, the Spearman correlation, Wilcoxon Signed-Ranked test, and Kruskal–Wallis H test. Pairwise comparisons were performed using Dunn’s procedure with a Bonferroni correction for multiple comparisons. All analysis was conducted at 0.05 level of significance. Urinary pesticide metabolite concentrations were compared across different maternal demographics and regions (Nickerie, Paramaribo, and the Interior) using non-parametric tests, the Spearman correlation, Wilcoxon Rank sum test, and Kruskal- Wallis H test, where assumptions of normality were not met. For parametric tests, we considered paired samples *t*-test, Pearson correlation or Spearman correlation, and one-way analysis of variance.

## 3. Results

### 3.1. Demographic Characteristics and General Pesticide Information

Demographic characteristics of the pregnant women are shown in Table 2. Urine samples were collected from 214 pregnant women residing in the districts of Paramaribo, Nickerie, and the Interior of Suriname. Ninety (42.1%) participants were from Paramaribo, 78 (36.4%) were from Nickerie, and 46 (21.5%) were from the Interior. The median age of the pregnant women was 27.5 years, and varied from 16.9 to 46.3 years. The majority of the women were more than 25 years of age (61.2%) and were of non-African descent (60.3%). The ethnic groups of the pregnant women were as follows: Hindustani (22.9%), Tribal Peoples (formerly known as Maroons) (20.6%), Creole (17.8%), Indigenous (15.9%), Javanese (10.7%), Mixed (10.3%) and Caucasian (0.5%). Most of the women were married or living with a partner (90.7%), received a secondary or tertiary education (72.4%), and had a household income of SRD < 3000 (equvalent to USD < 400 at the time) (70.6%). Lastly, 50.0% had a BMI of 25.0+ kg/m^2^ and were classified as overweight or obese. 

A chi- square test for association was conducted between location and the following demographic characteristics: age of the pregnant women (<25 years and 25+ years), education level, ethnicity, marital status, household income, and BMI. There was a statistically significant association between location and age groups of the pregnant women, (x^2^ = 7.22, *p* = 0.03). There was a statistically significant association between location and ethnicity (African descent and non-African descent; x^2^ = 53.03, *p* < 0.001). There was a statistically significant association between location and education (no or primary, lower vocational, lower secondary, upper secondary, and tertiary; x^2^ = 110.56, *p* < 0.001). There was a statistically significant association between location and marital status (married or living with partner and not married or not living with partner; x^2^ = 7.61, *p* = 0.02). There was a statistically significant association between location and BMI (<24.9 underweight or normal weight and 25.0+ overweight or obese; x^2^ = 6.85, *p* = 0.03). There was a statistically significant association between location and household income (SRD) (<3000 and 3000+; x^2^ = 16.95, *p* < 0.001).

The five pesticide metabolites with more than 60% of non-detectable concentrations are shown in the supplement as Table A1.

Successfully imputed pesticides and metabolites included 2,4-D, TCPy, IMPY, pNP, DEP, DMP, DMTP, DETP, and 3-PBA, as shown in Table A2. Median, 25th and 75th percentile, and range of concentrations along with the limits of detection (LOD) and the percentage of samples that were below the LOD are reported in Table A2.

### 3.2. Specific Gravity Measurements and Highly Correlated Metabolites

We had fresh specific gravity measurements for 45% of the participants. We were able to measure frozen specific gravity measurements for 93% of the participants. There were 91 participants who had specific gravity determined in both fresh and frozen samples. The Wilcoxon matched-pairs signed rank test was conducted between the fresh and frozen specific gravity measurements of the 91 pregnant women who had both measurements. No statistically significant difference was observed between median levels of the fresh and frozen specific gravity measurements (*p* = 0.36). The means of both the fresh and frozen specific gravity measurements were 1.02 μg/L. As a result, for all participants who were missing fresh specific gravity measurements, we used their frozen specific gravity measurements for urinary adjustments. We were missing specific gravity measurements for 6.5% of the participants (*n* = 14).

Descriptive statistics including minimum and maximum, 25th and 75th percentile, median and 95th confidence interval of the specific gravity adjusted pesticide and metabolite concentrations are found in Table 3.

**Figure 1:**
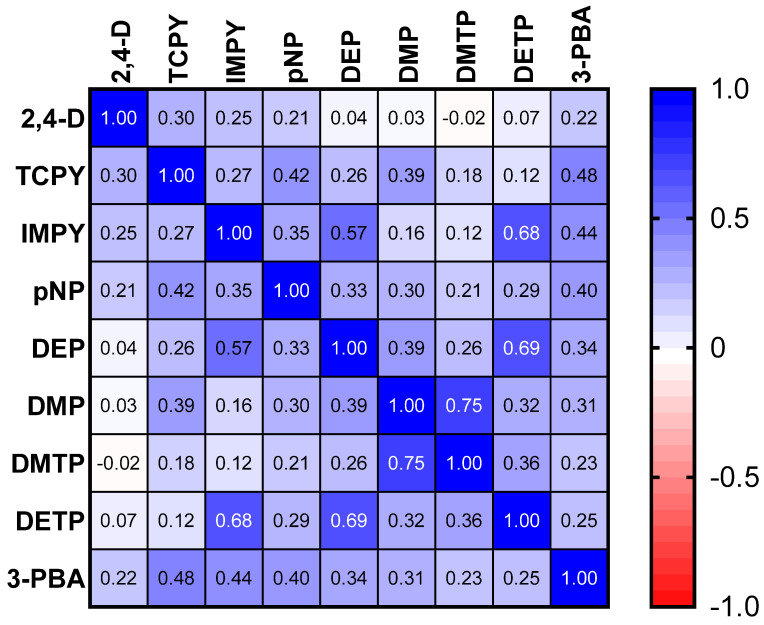
Spearman correlation matrix of specific gravity adjusted urinary pesticide metabolite concentrations.

**Figure 2:**
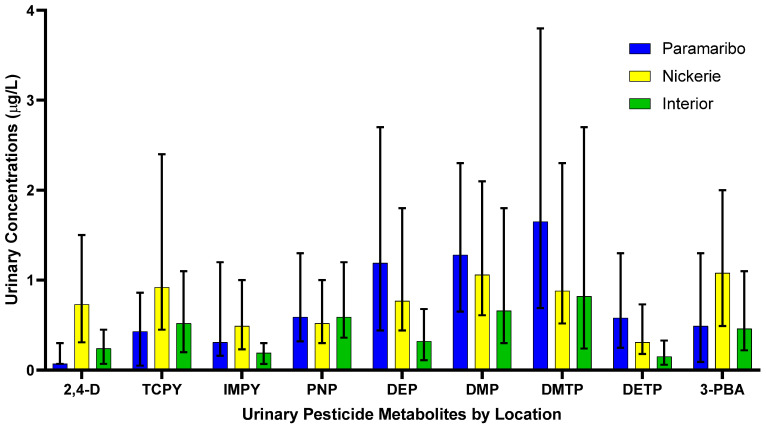
Specific gravity adjusted urinary pesticide metabolite concentrations from pregnant women in Suriname by location. Data is presented as median concentrations with error bars as the interquartile range.

The results of the spearman correlation of the specific gravity adjusted urinary pesticide metabolite concentrations indicate the monotonic relationships between the pesticide metabolites, which are showcased in Figure 1. There were strong, positive correlations between IMPY and DEP (r_s_ = 0.57), IMPY and DETP (r_s_ = 0.68), and DETP and DEP (r_s_ = 0.69), shown in Figure 1. Specific gravity adjusted urinary pesticide metabolite concentrations (SGAC) from pregnant women in Suriname across locations are presented in Figure 2.

In the following sections, we have provided the medians, the H value of the Kruskal- Wallis H test, and the *p*-value of the test results.

#### 3.2.1. Phenoxy Acid Herbicide

Pregnant women residing in Nickerie had significantly higher SGAC of 2,4-D compared to pregnant women residing in the Interior (0.30 μg/L; H = 50.52, *adj p* < 0.05) and those in Paramaribo (0.19 μg/L; H = −73.41, *p* < 0.05).

#### 3.2.2. Organophosphate Insecticides

The SGAC of TCPy between pregnant women residing in Paramaribo (0.44 μg/L) and the Interior (0.71 μg/L) (H = −30.87, *p* = 0.02), Paramaribo (0.44 μg/L) and Nickerie (1.26 μg/L) (H = −58.57, *p* < 0.05), and Nickerie (1.26 μg/L) and the Interior (0.71 μg/L) (H = 27.71, *p* = 0.04).

The SGAC of IMPY in pregnant women were significantly different between those in the Interior (0.27 μg/L) and Paramaribo (0.35 μg/L) (H = 32.87, *p* = 0.009), those in the Interior (0.27 μg/L) and Nickerie (0.60 μg/L) (H = 58.213, *p*< 0.05) and those in Nickerie (0.60 μg/L) and Paramaribo (0.35 μg/L) (H = −25.35, *p* = 0.02).

Pregnant women residing in the Interior (0.79 μg/L) had higher SGAC of of pNP compared to those in Paramaribo (0.78 μg/L), and in Nickerie (0.75 μg/L), but the distributions were not significantly different (H = 0.49, *p* = 0.782).

The SGAC of DEP were significantly lower among participants from the Interior (0.35 μg/L) compared with those from Paramaribo (1.50 μg/L) (H = 54.63, *p* < 0.05) and and Nickerie (1.19 μg/L) (H = 56.56, *adj p* < 0.05). Concentrations between Paramaribo (1.50 μg/L) and Nickerie (1.19 μg/L) were not statistically different (H = −1.94, *p* = 1.00).

The SGAC of DMP were statistically significantly different between women in the Interior (0.80 μg/L) and Nickerie (1.44 μg/L) (H = 33.51, *p* = 0.009) but not between pregnant women in Paramaribo (1.29 μg/L) and Nickerie (1.44 μg/L) (H = −9.36, *p* = 0.92), and those in the Interior (0.80 μg/L) and Paramaribo (1.29 μg/L) (H = 24.15, *p* = 0.09).

Pregnant women in Paramaribo (1.74 μg/L) had higher SGAC of DMTP compared to those in the Interior (1.28 μg/L) and Nickerie (1.36 μg/L), but the results were not significant (H = 2.43, *p* = 0.30). 

The SGAC of of DETP in pregnant women were significantly different between pregnant women in the Interior (0.27 μg/L) and Nickerie (0.44 μg/L) (H = 34.72, *p* = 0.006) and those in the Interior (0.27 μg/L) and Paramaribo (0.57 μg/L) (H = 48.30, *p* < 0.05) but not among those in Nickerie (0.44 μg/L) and Paramaribo (0.57 μg/L) (H = 13.61, *p* = 0.41).

#### 3.2.3. Pyrethroid Insecticide

The SGAC of the 3-PBA metabolite were significantly different between pregnant women in Paramaribo (0.65 μg/L) and Nickerie (1.34 μg/L) (H = −47.11, *p* < 0.05) and those in Nickerie (1.34 μg/L) and the Interior (0.70 μg/L) (H = 37.47, *p* = 0.003), but not in those residing in Paramaribo (0.65 μg/L) and the Interior (0.70 μg/L) (H = −9.65, *p* = 1.00).

### 3.3. Associations between Urinary Pesticide Metabolite Concentrations and Demographic Characteristics

The following results provide information of the associations of urinary pesticide metabolite concentrations and the demographic characteristics of the participants, shown in Table 4. There was a significant difference in 2,4-D concentrations of pregnant women in Nickerie and the different age groups, (z = 6.90, *p* = 0.009), with a mean rank concentration score of 45.03 for women <25 years and 31.70 for 25+ years. We observed a statistically significant difference in IMPY metabolite concentrations of pregnant women in Paramaribo and the different age groups, (z = 5.57, *p* = 0.018), with a mean rank concentration score of 33.12 for <25 years and 46.71 for 25+ years.

There was a significant difference in the 3-PBA concentrations of pregnant women in Paramaribo and the different ethnic groups, (z = 7.03, *p* = 0.008), with a mean rank concentration score of 36.82 for those who identified as being of African descent and 51.27 for those who identified as being of non-African descent. There was a significant difference in 2,4-D concentrations of pregnant women in the Interior and the different ethnic groups, (z = 3.90, *p* = 0.05), with a mean rank concentration score of 24.15 for those who identified as being of African descent and 16.85 for those who identified as being of non-African descent. There was a significant difference in IMPY concentrations of pregnant women in the Interior and the different ethnic groups, (z = 4.23, *p* = 0.04), with a mean rank concentration score of 24.30 for those who identified as being of African descent and 16.70 for those who identified as being of non-African descent. There was a significant difference in pNP concentrations of pregnant women in the Interior and the different ethnic groups, (z = 6.06, *p* = 0.01), with a mean rank concentration score of 25.05 for those who identified as being of African descent and 15.95 for those who identified as being of non-African descent. There was a significant difference in DTP concentrations of pregnant women in the Interior and the different ethnic groups, (z = 5.04, *p* = 0.03), with a mean rank concentration score of 24.65 for those who identified as being of African descent and 16.35 for those who identified as being of non-African descent.

There was a significant difference in IMPY concentrations in pregnant women in Paramaribo and household income, (z = 7.58, *p* = 0.006), with a mean rank of 35.25 for <3000 SRD and 50.25 for 3000+ SRD; between DEP concentrations and household income, (z = 6.31, *p* = 0.012), with a mean rank of 35.71 for <3000 SRD and 49.39 for 3000+ SRD; and between DTP concentrations and household income, (z = 6.41, *p* = 0.01), with a mean rank of 35.67 for <3000 SRD and 49.46 for 3000+ SRD. There was a significant difference DTP concentrations of pregnant women in Nickerie and household income, (z = 5.67, *p* = 0.017), with a mean rank of 32.22 for <3000 SRD and 45.00 for 3000+ SRD.

## 4. Discussion

This study was designed to assess exposures to select pesticides among pregnant women in the CCREOH cohort in Suriname. We provide the results of our analyses characterizing urinary concentrations of nine pesticide metabolites from the following chemical classes, phenoxy acid herbicides, organophosphate and pyrethroid insecticides among 214 pregnant women residing in both urban and rural districts in Suriname. The results indicate that pregnant women within the CCREOH cohort in Suriname are exposed to a phenoxy acid herbicide, 2,4-dichlorophenoxyacetic acid, multiple organophosphates insecticides, and multiple pyrethroid insecticides. Pregnant women residing in Nickerie had higher median urinary pesticide concentrations of 2,4- = D, TCPy, IMPY, and 3-PBA. The urinary concentrations of para-nitrophenol were higher among women residing in Paramaribo and in the Interior compared to those residing in Nickerie. The following dialkyl phosphates were highest among women living in Paramaribo compared to those residing in Nickerie and the Interior, diethylphosphate, dimethylphosphate, dimethylthiophosphate, and diethylthiophosphate.

Pesticide exposures differed significantly with respect to location, which aligns with the original hypothesis, in addition to age, ethnicity, and household income. High concentrations of 2,4-D among pregnant women residing in Nickerie could be contributed to the control for broadleaf weeds in both agricultural and residential settings and through diet [52]. Exposure to dialkyl phosphates in Paramaribo could potentially occur through diet. The main route of exposure to organophosphate insecticides is commonly through the ingestion of food, specifically of non- occupationally exposed individuals [53,54]. Individuals who live in an agricultural area, like Nickerie, are exposed to organophosphate insecticides through drinking water, air, and soil along with occupational exposure [55]. Previous research has found that higher intake of citrus fruits, apple juice, sweet peppers, tomatoes, beans and dry peas, soy and rice beverages, whole grain bread, white wine and green and herbal teas were significantly related to higher urinary DAP concentrations [54]. Additionally, exposure to these dialkyl phosphates could result from occupational exposure or the proximity to areas where there is heavy agricultural application of pesticides, which would primarily occur in Nickerie.

Higher urinary 3-PBA metabolite concentrations in the well-known agricultural district, like Nickerie could potentially be due to poor housing conditions of which are common in agricultural communities. This in turn could require an increase in the use of pesticides in order to control for pests [56,57,58,59]. In Suriname, mosquito coils, incense and candles are widely used to prevent from mosquito and bug bites and pyrethroids are the main ingredient in these products. Lastly, common household products, pet shampoos, and lice treatments are all products with pyrethroids as an ingredient which could potentially contribute to the pyrethroid exposure to pregnant women in Suriname [60].

There are numerous population studies that have documented urinary pesticide metabolite concentrations in pregnant women, including the Generation R study, the Center for the Health Assessment of Mothers and Children of Salinas (CHAMACOS) study, and the National Health and Nutrition Examination Survey (NHANES). The Generation R study is a population-based prospective cohort study conducted in Rotterdam, Netherlands [61]. Pregnant women in the Generation R cohort study had higher geometric means concentrations of DMP and DMTP compared to women residing in Paramaribo, Nickerie, and the Interior [62]. The CHAMACOS study, a longitudinal birth cohort study of pesticides and other environmental exposures among children in a large farmworker community in Salinas Valley, California investigated the organophosphate creatinine-adjusted urinary metabolite levels during pregnant and after delivery in women residing in the an agricultural community. We found that in the CCREOH cohort, pregnant women residing in Nickerie, an agricultural community, had lower geometric mean concentrations of DEP, DETP, DMP, and DMTP compared to those from the CHAMACOS study [58]. We also compared the urinary pesticide metabolite levels of organophosphate insecticides of pregnant women in the CCREOH cohort to women in the U.S. population utilizing the NHANES in 2003–2004. Pregnant women in the U.S. population had higher geometric mean concentrations of DMTP compared to pregnant women residing in Paramaribo, Nickerie and the Interior [63].

Lastly, we compared the urinary metabolite concentrations of organophosphate insecticides and phenoxy acid herbicides of pregnant women in Suriname to pregnant women residing from 10 Caribbean countries, Antigua and Barbuda, Belize, Bermuda, Dominica, Grenada, Jamaica, Montserrat, St. Kitts and Nevis, St. Lucia, and St. Vincent and the Grenadines [64]. Pregnant women in Suriname had lower geometric means of urinary metabolite concentrations of DEP compared to those in Antigua and Barbuda, Bermuda, Dominica, Grenada, Jamaica, and St. Kitts and Nevis [64]. Geometric means of the urinary metabolite concentrations of DETP in pregnant women in Nickerie were similar to pregnant women living in St. Vincent and the Grenadines, which is understandable as the country’s economy is based heavily on agriculture [64]. Pregnant women residing in Antigua and Barbuda, Bermuda, Grenada, St. Lucia, St. Kitts, and St. Vincent had higher geometric means of urinary metabolite concentrations of DMP compared to those living in Suriname [65]. Lastly, pregnant women residing in the Interior and Paramaribo had similar geometric means of urinary metabolite concentrations of 2,4-D to pregnant women living in Bermuda and Dominica [65]. The differences in dialkyphosphates observed in the various countries could be due to the differing roles of women in agricultural work, different agricultural practices and cultural contexts, and differential regulatory standards on protective personal equipment.

For the pyrethroid, 3-phenoxybenzoic acid (3-PBA), pregnant women residing in Nickerie (1.03 μg/L) had a higher geometric mean concentration compared to those residing in Belize (0.21 μg/L), Bermuda (0.56 μg/L), Dominica (0.45 μg/L), Grenada (0.81 μg/L), Jamaica (0.32 μg/L), Montserrat (0.36 μg/L), St. Lucia (0.58 μg/L), St. Kitts and Nevis (0.64 μg/L), St. Vincent and the Grenadines (0.54 μg/L), except for pregnant women residing in Antigua and Barbuda (1.77 μg/L), which could be due to the demand of agricultural production in Nickerie [66].

Strengths of this study include the prospective study design focusing on a vulnerable population in Suriname that had not been assessed for pesticide exposure previously. We adjusted the data for specific gravity compared to creatinine adjusted. Creatinine adjusted data is the traditional approach, however, specific gravity is a simpler and less cost intensive approach [67]. It has been found that creatinine and specific gravity adjusted measurements are equivalent correct factors. As a result, the use of specific gravity adjusted measurements did not change or affect the observed associations [67]. This is the first baseline assessment of urinary pesticide metabolite concentrations among pregnant women conducted in Suriname. We hope this study will provide the evidence base for the development of effective pesticide policies in Suriname, and to create and implement effective intervention strategies that will aid in reducing pesticide exposure among pregnant women in the country. This study had a few limitations, which include the lack of demographic data for all participants, exposure misclassification, and given the one time urine collection, not being able to account for potential seasonal variability. Demographic data for a few women who participated in the study were missing due to technological issues in the field. However, we imputed missing data and were able to observe associations between the demographic characteristics and specific pesticide metabolites with our final study population. We assessed at one time cross-sectionally, however, urinary pesticide concentrations could have temporal and within-individual variability. Additionally, due to the temporal relationship of pesticide exposure, we may have only captured acute exposure of pesticides. Future research directions include the collection of multiple spot urine samples at various time points during pregnancy. Due to the various seasons in Suriname, seasonal variation in pesticide exposures is anticipated due to different pesticides used during specific seasons. Other researchers have reported seasonal variability in pesticide exposures while examining numerous urinary pesticide metabolites throughout an agricultural season in Latino farmworkers in the US [57].

## 5. Conclusions

This is the first baseline study documenting exposures to herbicide and insecticide pesticides in pregnant women in Suriname using urinary biomarkers. Populations from three regions of the country were included. This study observed differences in pesticide exposures among the geographic regions, which is most likely related to the use of different pesticides for different purposes. Additional research is necessary to determine if there is a relationship between longitudinally measured pesticide exposures and adverse health outcomes.

## Figures and Tables

**Table 1:** Chemical Class and Associated Parent Chemical.

Analyte, Analyte Abbreviation	Parent Chemical
**Chemical Class: Phenoxyacetic Acid Herbicides**	
2,4-dichlorophenoxyacetic acid, 2,4-D	2,4-dichlorophenoxyacetic acid (and its esters)
**Chemical Class: Organophosphate Insecticides**	
Malathion dicarboxylic acid, MDA	Malathion
3,5,6-trichloro-2-pyridinol, TCPy	Chlorpyrifos, chlorpyrifos-methyl
2-isopropyl-4-methyl-6-hydroxypyrimidine, IMPY	Diazinon
para-nitrophenol, pNP	Parathion, Methyl parathion
Diethyldithiophosphate, DEDTP	
Diethylphosphate, DEP	
Dimethyldithiophosphate, DMDTP	
Dimethylphosphate, DMP	
Dimethylthiophosphate, DMTP	
Diethylthiophosphate, DETP	
**Chemical Class: Pyrethroid Insecticides**	
4-fluoro-3-phenoxybenzoic acid, 4-F-3-PBA	Cyfluthrin, Flumethrin
3-phenoxybenzoic acid, 3-PBA	Cyhalothrin, cypermethrin, deltamethrin, fenpropathrin, permethrin, tralomethrin, allethrin, resmethrin, fenvalerate, lambda-cyhalothrin, D-phenothrin, fenpropathrin
trans-3-(2,2-dichlorovinyl)-2,2-dimethylcyclopropane carboxylic acid, *trans*-DCCA	Permethrin, Cypermethrin, Cyfluthrin

**Table 2:** Demographic characteristics of the pregnant women in Suriname, *n* = 214.

Characteristics	Overall (*n* = 214) *n* (%)	Paramaribo (*n* = 90) *n* (%)	Nickerie (*n* = 78) *n* (%)	Interior (*n* = 46) *n* (%)
Age (in years) ^1^				
<25 years	80 (37.4)	26 (28.9)	30 (38.5)	24 (52.2)
25+ years	131 (61.2)	64 (71.1)	45 (57.7)	22 (47.8)
Ethnicity ^2^				
African descent	82 (38.3)	55 (61.1)	5 (6.4)	22 (47.8)
Non-African descent	129 (60.3)	35 (38.9)	70 (89.7)	24 (52.2)
Education ^2^				
No or primary	56 (26.2)	13 (14.4)	4 (5.1)	39 (84.8)
Lower vocational	40 (18.7)	22 (24.4)	13 (16.7)	5 (10.9)
Lower secondary	28 (13.1)	11 (12.2)	15 (19.2)	2 (4.3)
Upper secondary	66 (30.8)	33 (35.6)	35 (43.6)	
Tertiary	21 (9.8)	12 (13.3)	9 (11.5)	
Marital Status ^1^				
Not married or not living with partner	17 (7.9)	12 (13.3)	5 (6.4)	
Married or living with partner	194 (90.7)	78 (86.7)	70 (89.7)	46 (100.0)
BMI ^1^				
<24.9 underweight or normal weight	87 (40.7)	26 (28.9)	35 (44.9)	26 (56.5)
25.0+ overweight or obese	107 (50.0)	49 (54.5)	40 (51.3)	18 (39.1)
Household income ^2^				
<3000	151 (70.6)	56 (62.2)	52 (66.7)	43 (93.5)
3000+	53 (24.8)	30 (33.3)	22 (28.2)	1 (2.2)

SRD-Surinamese dollars. ^1^ The statistically significant association between location and the specific demographic characteristic, *p* < 0.05. ^2^ The statistically significant association between location and the specific demographic characteristic, *p* < 0.001.

**Table 3:** Specific gravity adjusted urinary pesticide and metabolite concentrations (μg/L) in pregnant women in Suriname by location, *n* = 214.

Analyte Code	LOD	Location	Standard Deviation	Min	25th Percentile	Median (95% CI)	75th Percentile	Max
2,4-D	0.15 ^1,2^	Paramaribo	0.38	<LOD	<LOD	0.19, (0.22, 0.38)	0.34	2.12
		Nickerie	2.11	<LOD	0.46	1.06, (1.12, 2.09)	1.69	10.88
		Interior	1.16	<LOD	<LOD	0.30, (0.24, 0.98)	0.66	7.04
TCPy	0.10 ^1,2,3^	Paramaribo	0.59	<LOD	0.13	0.44, (0.44, 0.70)	0.75	2.83
		Nickerie	2.84	<LOD	0.65	1.26, (1.80, 3.11)	3.06	13.69
		Interior	5.71	<LOD	0.32	0.71, (0.36, 4.01)	1.14	34.59
IMPY	0.10 ^1,2,3^	Paramaribo	2.89	<LOD	0.21	0.35, (0.75, 2.00)	0.96	16.92
		Nickerie	5.04	<LOD	0.37	0.60, (0.58, 2.90)	1.58	43.33
		Interior	0.30	<LOD	<LOD	0.27, (0.21, 0.40)	0.41	1.69
pNP	0.10	Paramaribo	0.72	<LOD	0.43	0.78, (0.79, 1.10)	1.29	5.03
		Nickerie	1.96	0.21	0.46	0.75, (0.86, 1.76)	1.43	11.66
		Interior	10.40	0.25	0.56	0.79, (−0.73, 5.92)	1.25	66.64
DEP	0.10 ^2,3^	Paramaribo	2.24	<LOD	0.50	1.50, 1.57, 2.54)	2.71	12.42
		Nickerie	6.90	<LOD	0.59	1.19, (1.36, 4.54)	2.25	43.75
		Interior	0.86	<LOD	0.15	0.35, (0.35, 0.90)	0.57	4.18
DMP	0.10 ^2^	Paramaribo	3.28	<LOD	0.75	1.29, 1.38, 2.81)	2.28	28.33
		Nickerie	1.97	0.24	0.90	1.44, (1.65, 2.56)	2.48	12.77
		Interior	2.88	0.08	0.49	0.80, (1.01, 2.85)	1.87	13.51
DMTP	0.10	Paramaribo	3.87	<LOD	0.72	1.74, (2.40, 4.07)	4.12	20.43
		Nickerie	3.78	0.14	0.70	1.36, (1.97, 3.71)	3.19	19.23
		Interior	5.72	<LOD	0.41	1.28, (1.34, 5.00)	2.99	25.82
DETP	0.10 ^2,3^	Paramaribo	2.97	<LOD	0.29	0.57, (0.88, 2.17)	1.42	20.08
		Nickerie	5.57	<LOD	0.23	0.44, (0.10, 2.67)	0.89	48.50
		Interior	0.37	<LOD	0.11	0.27, (0.24, 0.48)	0.45	1.85
3-PBA	0.10 ^1,2^	Paramaribo	1.15	<LOD	0.26	0.65, (0.67, 1.17)	1.13	6.80
		Nickerie	7.06	0.14	0.65	1.34, (1.58, 4.83)	2.41	53.37
		Interior	0.85	<LOD	0.31	0.70, (0.70, 1.24)	1.38	3.53

<LOD–Less than the limit of detection. ^1^ Statistically significant difference between Nickerie and Paramaribo, *p* < 0.05. ^2^ Statistically significant difference between Nickerie and the Interior, *p* < 0.05. ^3^ Statistically significant difference between Paramaribo and the Interior, *p* < 0.05.

**Table 4:** Associations between urinary pesticide metabolite concentrations and demographic characteristics.

Characteristics	Analyte Code	Mean Rank Concentration Score
**Age Groups**		
<25 years	2,4-D ^2^	45.03 ^†^
25+ years		31.70
<25 years	IMPY ^1^	33.12 *
25+ years		46.71
**Ethnic Groups**		
African descent	3-PBA ^1^	36.82
non-African descent		51.27
African descent	2,4-D ^3^	24.15 *
non-African descent		16.85
African descent	IMPY	24.30
non-African descent		16.70
African descent	PNP	25.05
non-African descent		15.95
African descent	DTP	24.65
non-African descent		16.35
**Household Income**		
<3000 SRD	IMPY ^1^	35.25 ^†^
3000+ SRD		50.25
<3000 SRD	DEP ^1^	35.71 *
3000+ SRD		49.39
<3000 SRD	DTP^1^	35.67 ^†^
3000+ SRD		49.46
<3000 SRD	DTP^2^	32.22 *
3000+ SRD		45.00

^1^ Paramaribo; ^2^ Nickerie; ^3^ Interior; * p < 0.05; ^†^ *p* < 0.01.

## Data Availability

The data presented in this study can be made available based on a reasonable request. Such requests should be directed to the PI’s of the Caribbean Consortium for Research in Environmental and Occupational Health (CCREOH) through the intermediary of the corresponding author.

## Appendix A

**Table A1:** Descriptive statistics of pesticide metabolites with over 60% of results below the limit of detection.

Analyte	LOD (μg/L)	% Below LOD	25th Percentile (μg/L)	Median (μg/L)	75th Percentile (μg/L)	Range (μg/L)
**Chemical Class: Organophosphate insecticides**						
Malathion dicarboxylic acid, MDCA	0.50	75.94	<LOD	<LOD	<LOD	<LOD-51.68
Diethyldithiophosphate, DEDTP	0.10	98.58	<LOD	<LOD	<LOD	<LOD-0.14
Dimethyldithiophosphate, DMDTP	0.10	72.17	<LOD	<LOD	0.12	<LOD-11.70
**Chemical Class: Pyrethroid insecticides**						
4-fluoro-3-phenoxybenzoic acid, 4F-3-PBA	0.10	98.11	<LOD	<LOD	<LOD	<LOD-0.29
trans-3-(2,2-dichlorovinyl)-2,2-dimethylcyclopropane carboxylic acid, Trans-DCCA	0.60	60.38	<LOD	<LOD	1.56	<LOD-27.20

LOD-Limit of detection.

**Table A2:** Descriptive statistics of pesticide metabolites.

Analyte	LOD (μg/L)	% Below LOD	25th Percentile (μg/L)	Median (μg/L)	75th Percentile (μg/L)	Range (μg/L)
**Chemical Class: Phenoxy acid herbicide**						
2, 4- Dichlorophenoxyacetic acid, 2,4-D	0.15	35.38	<LOD	0.29	0.73	<LOD-9.00
**Chemical Class: Organophosphate insecticide**						
3,5,6-Trichloro-2-pyridinol, TCPy	0.10	22.64	0.17	0.59	1.17	<LOD-46.70
2-isopropyl-4-methyl-6-hydroxypyrimidine, IMPY	0.10	16.51	0.16	0.31	0.74	<LOD-52.00
para-Nitrophenol, pNP	0.10	0.94	0.31	0.58	1.17	<LOD-73.30
Diethylphosphate, DEP	0.10	12.26	0.33	0.76	1.93	0–52.50
Dimethylphosphate, DMP	0.10	3.77	0.56	1.05	2.06	<LOD-42.50
Dimethylthiophosphate, DMTP	0.10	5.19	0.51	1.19	3.14	<LOD-25.00
Diethylthiophosphate, DETP	0.10	18.40	0.17	0.34	0.86	0–58.20
**Chemical Class: Pyrethroid insecticide**						
3-phenoxybenzoic acid, 3-PBA	0.10	15.57	0.20	0.72	1.55	0–50.70

LOD-Limit of Detection.
